# The Division of Fees

**Published:** 1910-04

**Authors:** 


### The Division of Fees

AT the February meeting of the Medical Society of the County of Erie unusual interest was created, through the presentation of a paper by Dr. M. D. Mann, relating to the dividing of fees between the consulting and the attending physician or surgeon. This topic has been discussed or talked of at low breath for many years; indeed, sometimes it has been a subject of acrimonious debate between individuals. In the medical journals, too, it has now and then been touched upon, for the most part, however, in a mild or at least, in an imperfect manner, because dreading apparently to give it the publicity it deserves, or to deal with it in such fashion as the evil merits. But it has remained for Dr. Mann to handle the viper without gloves and to hold it up to view, as though intending to cast it out from a profession already bitten too severely by its poisonous fangs.

This courageous and masterful paper will be found in the original department of this number and we trust it will be read in full by physicians in all parts of the world. The proceedings of the society, too, are published in this issue, whereby it may be noted that a rising vote of approbation was given the author. To further emphasise its approval the society ordered 1,000 copies distributed to physicians in this vicinity. We feel sure this marks the beginning of a campaign that will put an end to this nefarious practice in this region at least.

